# Association of *ABCC2* polymorphism with clopidogrel response in Chinese patients undergoing percutaneous coronary intervention

**DOI:** 10.3389/fphar.2022.889473

**Published:** 2022-10-07

**Authors:** Lida Chen, Chao Zheng, Mengmeng Hao, Peng Gao, Meimei Zhao, Yongtong Cao, Liang Ma

**Affiliations:** ^1^ Department of Blood Transfusion, China-Japan Friendship Hospital, Beijing, China; ^2^ Department of Clinical Laboratory, China-Japan Friendship Hospital, Beijing, China

**Keywords:** *ABCC2* polymorphism, clopidogrel response, percutaneous coronary intervention, individualized difference, precise medication

## Abstract

**Aim:** In this study, we investigated the association between *ABCC2* polymorphism and clopidogrel response as well as the associated hypothetical mechanism.

**Methods:** Chinese patients (213) with coronary artery disease (CAD) who underwent percutaneous coronary intervention (PCI) and received clopidogrel were recruited. Thereafter, their ADP-induced platelet inhibition rates (PAIR%) were determined *via* thromboelastometry. Further, the single-nucleotide polymorphisms (SNPs) of *ABCC2* were genotyped using high-resolution melting curve (HRM)-PCR, while CYP2C19*2 and *3 polymorphisms were genotyped *via* real-time PCR.

**Results:** The allele frequencies of *ABCC2* rs717620 were 74.88 and 25.12% for the C and T alleles, respectively. Further, *ABCC2* rs717620 TT carriers exhibited significantly higher PAIR% values (72.60 ± 27.69) than both CT (61.44 ± 23.65) and CC carriers (52.72 ± 21.99) (*p* = 0.047 and *p* = 0.001, respectively), and *ABCC2* rs717620 CT carriers showed significantly higher mean PAIR% values than *ABCC2* rs717620 CC carriers (*p* = 0.011). However, the PAIR% values corresponding to *ABCC2* rs2273697 and *ABCC2* rs3740066 carriers were not different. Additionally, CYP2C19*2 AA carriers presented significantly lower PAIR% values than CYP2C19*2 GA (*p* = 0.015) and GG (*p* = 0.003) carriers, and CYP2C19*3 GA carriers also presented significantly lower PAIR% values than CYP2C19*3 GG carriers (*p* = 0.041). In patients with *CYP2C19* extensive metabolizers (EM), *ABCC2* rs717620 TT carriers showed significantly higher PAIR% values (89.77 ± 9.73) than CT (76.76 ± 26.00) and CC carriers (74.09 ± 25.29) (*p* = 0.040 and *p* = 0.009, respectively). In patients with *CYP2C19* poor metabolizers (PM), *ABCC2* rs717620 CC carriers showed significantly lower PAIR% values (51.72 ± 25.78) than CT carriers (75.37 ± 23.57) (*p* = 0.043). Furthermore, after adjusting for confounding factors, *ABCC2* rs717620 was identified as a strong predictor of clopidogrel hyperreactivity.

**Conclusion:** We proposed a new target, *ABCC2* rs717620, in the efflux pathway that affects individual responses to clopidogrel. The TT allele of *ABCC2* rs717620 was also identified as an independent risk factor for clopidogrel hyperreactivity, and CYP2C19*2 and *3 showed association with an increased risk for clopidogrel resistance. Additionally, *ABCC2* rs717620 may affect individual responses to clopidogrel *via* post-transcriptional regulation and interaction with *CYP2C19*. These findings provide new insights that may guide the accurate use of clopidogrel.

## Introduction

Clopidogrel is an antiplatelet agent that is widely used in patients with coronary artery disease (CAD) undergoing percutaneous coronary intervention (PCI) (Watanabe et al., 2019). However, pharmacodynamic responses to clopidogrel differ significantly between individuals (Akkaif et al., 2021). Further, *CYP2C19* plays a vital role in clopidogrel transformation; thus, the Food and Drug Administration (FDA) recommends that tests be conducted for its identification prior to the commencement of clopidogrel treatment (Holmes et al., 2010). Specifically, clopidogrel shows high activity in patients with fast *CYP2C19* metabolism (EM), while those with poor *CYP2C19* metabolism (PM) often show clopidogrel resistance (Bolcato et al., 2021). Paradoxically, some studies have revealed that some patients with EM-type *CYP2C19* show clopidogrel resistance rates as high as 34.6% ± 0.6% (Zhang et al., 2018), while 15.8% of patients with PM-type *CYP2C19* experience bleeding events caused by clopidogrel hyperreactivity (Nagashima et al., 2013). Further, it has been observed that only 15% of clopidogrel is metabolized, while up to 85% is excreted, but *CYP2C19* is only responsible for the metabolism of this drug (Simon et al., 2009). Thus, when *CYP2C19* is the only factor considered to guide clopidogrel treatment, there remains a risk of bleeding or thrombosis. Furthermore, it is still unclear whether targets in other pathways are involved in individual responses to clopidogrel. Interestingly, the efflux pathway for clopidogrel has rarely been reported. ABCC2, which is an ATP binding cassette efflux transporter that belongs to the ATP-binding cassette (ABC) superfamily of transmembrane proteins, is primarily expressed in polarized cells, such as hepatocytes, intestinal epithelia, and renal proximal tubule cells (Zhu et al., 2022). Specifically, ABCC2 plays an important role in the use of ATP hydrolysis energy to transfer its binding substrate out of the plasma membrane and mediate the transmembrane transport of several exogenous and endogenous drug metabolites. Thus, altered ABCC2 functioning owing to *ABCC2* single nucleotide polymorphisms (SNPs) can change the clearance, organ distribution, and absorption of several clinically important drugs, including antibiotics (e.g., ceftriaxone, rifampicin, and ampicillin), toxins, anti-hyperlipidemia inhibitors, and several cancer chemotherapy drugs (e.g., MTX, vinblastine, and irinotecan) (Jemnitz et al., 2010). *ABCC2* rs717620, rs2273697, and rs3740066, are the three common SNP genotypes of *ABCC2* in East-Asian (Han Chinese) ethnic population according to the SNP database of 1000 Genomes Project Database (www.1000genomes.org). These three SNPs are related to several clinical functions. For example, *ABCC2* rs717620 is related to the low effect of simvastatin treatment on HDL-C level in Chinese Han population (Liu et al., 2018). Further, in the Asia Pacific epilepsy cohort, *ABCC2* rs2273697 and rs3740066 polymorphisms were associated with antiepileptic drug resistance (Sha’Ari et al., 2014). It has also been observed that *ABCC2* rs3740066 is significantly associated with the vomiting of docetaxel in patients with breast cancer (Jabir et al., 2018). Therefore, in this study, these three SNPs were selected for further research.

Interestingly, the relationships between *ABCC2* gene polymorphisms, clopidogrel reactivity, and individual responses to clopidogrel have not yet been reported. Therefore, we investigated the effect of *ABCC2* gene polymorphisms on the variability of responses to clopidogrel and the possible associated mechanisms. The results thus obtained will provide insights into the effect of *ABCC2* polymorphisms on clopidogrel precision medicine, and the clarification of the possible associated mechanisms will also provide new ideas for the efflux of other *ABCC2*-dependent drugs in other diseases.

## Materials methods

### Study patients

Blood samples were collected from 213 hospitalized patients with CAD aged 20–75 years, who underwent PCI between January 2020 and December 2021. Following their first PCI for CAD at the Department of Cardiology of the China-Japan Friendship Hospital, the patients received a daily maintenance clopidogrel dose of 75 mg for 7 days. Finally, samples were collected 6 h after the administration of the treatment on Day 7. Patients with liver, kidney, or lung failure, malignant tumors or hemorrhagic diseases were excluded. The study protocol was reviewed and approved by the Clinical Research Ethics Committee of the China-Japan Friendship Hospital (No. 2019-96-K64). All the study procedures were performed in accordance with the principles set forth in the Declaration of Helsinki.

### Determination of clopidogrel reactivity

The clopidogrel reactivity levels corresponding to the subjects were assessed using the TEG 5000 Thrombelastograph^®^ Hemostasis Analyzer System (Haemoscope; Haemonetics Corporation, Braintree, MA, United States) and expressed as ADP-induced platelet inhibition rates (PAIR%). Further, on Day 7 after the PCI, maximal amplitudes (MA)-ADP, R values (coagulation reaction time), K angle (coagulation time), and coagulation indexes (CIs) were determined using the TEG 5000 system, and ADP% values were calculated using a software based on the following expression: PAIR (%) = $\left(1-\frac{{MA}_{ADP}-{MA}_{fibrin}}{{MA}_{thrombin}-{MA}_{fibrin}}\right)\times 100$ . Furthermore, the PAIR values obtained were also used to assess platelet reactivity. Based on Wang et al. (2019), patients with PAIR ≤30% were classified as the high platelet reactivity (HPR) group (clopidogrel resistance), while those with PAIR >30% were classified as the low platelet reactivity (LPR) group (clopidogrel hyperreactivity) (HPR, PAIR ≤30%; LPR, PAIR >30%).

### DNA extraction and genotyping

Genomic DNA was extracted from ethylenediamine tetra acetic acid (EDTA)-anticoagulated peripheral blood (4 ml) using the Thermo Kingfisher Flex system (BOKUN BIOTECH, Changchun, China) according to the manufacturer’s instructions and stored at −40°C until further analysis.

(1) Detection of *ABCC2* gene polymorphism and genotyping

A high-resolution melting curve (HRM)-PCR *ABCC2* genotyping assay was developed to detect *ABCC2* gene polymorphisms. The detection assay is detailed in the Chinese patent application: “A primer design method and kit for the detection of human ABCC2 gene polymorphism” (CN Patent number ZL 201910110953.6). The HRM-PCR was performed with our *ABCC2* genotyping assay using a LightCycler^®^ 480 instrument (Roche, Basel, Switzerland) to detect the three common SNP genotypes of *ABCC2* (rs717620, rs2273697, and rs3740066) in East-Asian ethnic population, as described in the introduction section. The genotypes of these three SNPs can be clearly and rapidly distinguished using this method, which is consistent with the sequence typing results.

(2) Determination of *CYP2C19* gene polymorphisms and genotyping

Genetic testing for *CYP2C19* revealed the alleles CYP2C19*1, CYP2C19*2 (variants in exons 2 and 5) and CYP2C19*3 (variants in exon 4) (Khaliq et al., 2000). Using the procedure of de De Morais et al. (1994) genomic DNA was extracted, amplified, and analyzed by restriction fragment length. In brief, the patients were divided into three metabolic groups based on *CYP2C19* genotypes (*CYP2C19* *1 [wild type], CYP2C19*2 rs4244285 [c.681G > A], and CYP2C19*3 rs4986893 [c.636G > A]), as follows: extensive metabolizers (EM; CYP2C19*1/*1), intermediate metabolizers (IM; CYP2C19*1/*2 or *1/*3), and poor metabolizers (PM; CYP2C19*2/*2 or *3/*3).

### Statistical analysis

Statistical analyses were performed using SPSS software version 20.0 (IBM Corp, Armonk, NY, United States). Categorical data were summarized as counts (percentages), while continuous variables were expressed as the mean ± standard deviation (SD). The power to calculate sample size was estimated by the PS-Power Sample Size software (version 3.1.2). Two-group comparisons were performed by conducting the Wilcoxon rank-sum test or χ2 test, when appropriate. Deviation from Hardy-Weinberg equilibrium (HWE) for *ABCC2* and *CYP2C19* SNPs was assessed using the χ2 tests. Further, a stepwise multiple linear regression analysis was performed to assess the most appropriate SNP parameter combination for estimating PAIR%. A multivariate logistic regression model was also constructed to adjust for important confounders and to assess clopidogrel resistance/hyperreactivity. Effect size estimates were provided as odds ratios (ORs) and 95% CIs. All the statistical tests were two-tailed, and *p* values <0.05 were considered statistically significant.

### *ABCC2* rs717620 mRNA secondary structure prediction

We have used the RNA structure software (http://rna.urmc.rochester.edu/RNAstructureWeb/) to predict the effect of the rs717620 mutant allele on the RNA secondary structure of *ABCC2*. Using the *ABCC2* rs717620 5′-UTR mRNA sequence as the input, the predicted secondary structure was verified to ensure that it has a negative minimum folding free energy (MFE) and a high minimum folding free energy index (MFEI), and the optimal mRNA 5′-UTR stem-loop secondary structures for wild-type and mutant *ABCC2* rs717620 served as output.

## Results

### Patient characteristics

Overall, 213 patients with CAD who underwent PCI were recruited, and their baseline characteristics are shown in Table 1. Basically, 211 (99.06%) of the patients were Han Chinese. Further, there were 23 (10.8%) patients in the HPR group and 190 (89.2%) in the LPR group, and the LPR group showed significantly higher high-density lipoprotein-cholesterol (HDL-C) levels than the HPR group (*p* = 0.029). Furthermore, there were no significant differences between the two groups with respect to sex, age, ethnicity (Han Chinese), platelet count (PLT), mean platelet volume (MPV), homocysteine (HCY) levels, total cholesterol (CHO) levels, triglyceride (TG) levels, or low-density lipoprotein cholesterol (LDL-C) levels.

**TABLE 1:** Demographic characteristics of patients.

Characteristics (*n* = 213)	HPR (*n* = 23)	LPR (*n* = 190)	p^a^ value
Male, n (%)	17 (73.91%)	146 (76.84%)	0.491
Age (years)	61.14 ± 14.54	62.45 ± 12.33	0.689
Ethnicity (Han Chinese), n (%)	22 (95.65%)	189 (99.47%)	0.205
PLT (109/L)	210.5 ± 81.43	226.8 ± 66.13	0.375
MPV (fL)	10.29 ± 1.35	10.24 ± 2.69	0.874
HCY (μmol/L)	15.11 ± 8.17	15.99 ± 9.45	0.670
CHO (mmol/L)	4.85 ± 1.13	4.68 ± 1.19	0.529
TG (mmol/L)	2.50 ± 1.46	1.80 ± 1.31	0.090
HDL-C (mmol/L)	0.87 ± 0.28	1.02 ± 0.35	**0.029**
LDL-C (mmol/L)	3.09 ± 1.09	2.98 ± 0.97	0.649

The bold value means *p* < 0.05, which was considered statistically significant.

a Comparison between the HPR, and LPR, groups; PLT, platelet count; MPV, mean platelet volume; HCY, homocysteine; CHO, total cholesterol; TG, triglyceride; HDL-C, high-density lipoprotein cholesterol; LDL-C, low-density lipoprotein cholesterol; PAIR (%), ADP-induced platelet aggregation inhibition rate; LPR: PAIR >30%, low platelet reactivity; HPR: PAIR ≤30%, high platelet reactivity.

### Influence of the single-nucleotide polymorphisms of *ABCC2* and *CYP2C19* on PAIR% during clopidogrel therapy

The allele frequencies of all the five SNPs (*ABCC2* rs717620, rs2273697, rs3740066, CYP2C19*2, and CYP2C19*3) in the patients were in accordance with the Hardy-Weinberg equilibrium (HWE). In *ABCC2* rs717620, the allele frequencies were 74.88 and 25.12% for C- and T-alleles, respectively. In CYP2C19*2, they were 74.41 and 25.59% for G- and A-alleles, respectively, and in CYP2C19*3, they were 93.90 and 6.10% for G- and A-alleles, respectively. Frequency comparison with East-Asian (Han Chinese) reference subpopulations was shown in Supplement Table S1, indicated no significant difference between our study and reference subpopulations. The *p*-values of all the five SNPs (*ABCC2* rs717620, rs2273697, rs3740066, CYP2C19*2, and CYP2C19*3) were 0.248, 0.123, 0.929, 0.117, 0.753, respectively, indicating that all the allele frequencies of *ABCC2* rs717620, rs2273697, rs3740066, CYP2C19*2 (rs4244285), and *CYP2C19* *3 (rs4986893) were consistent with the known East-Asian (Han Chinese) ethnic frequency reported in the 1000 Genomes Project Database (www.1000genomes.org).

**FIGURE 1:**
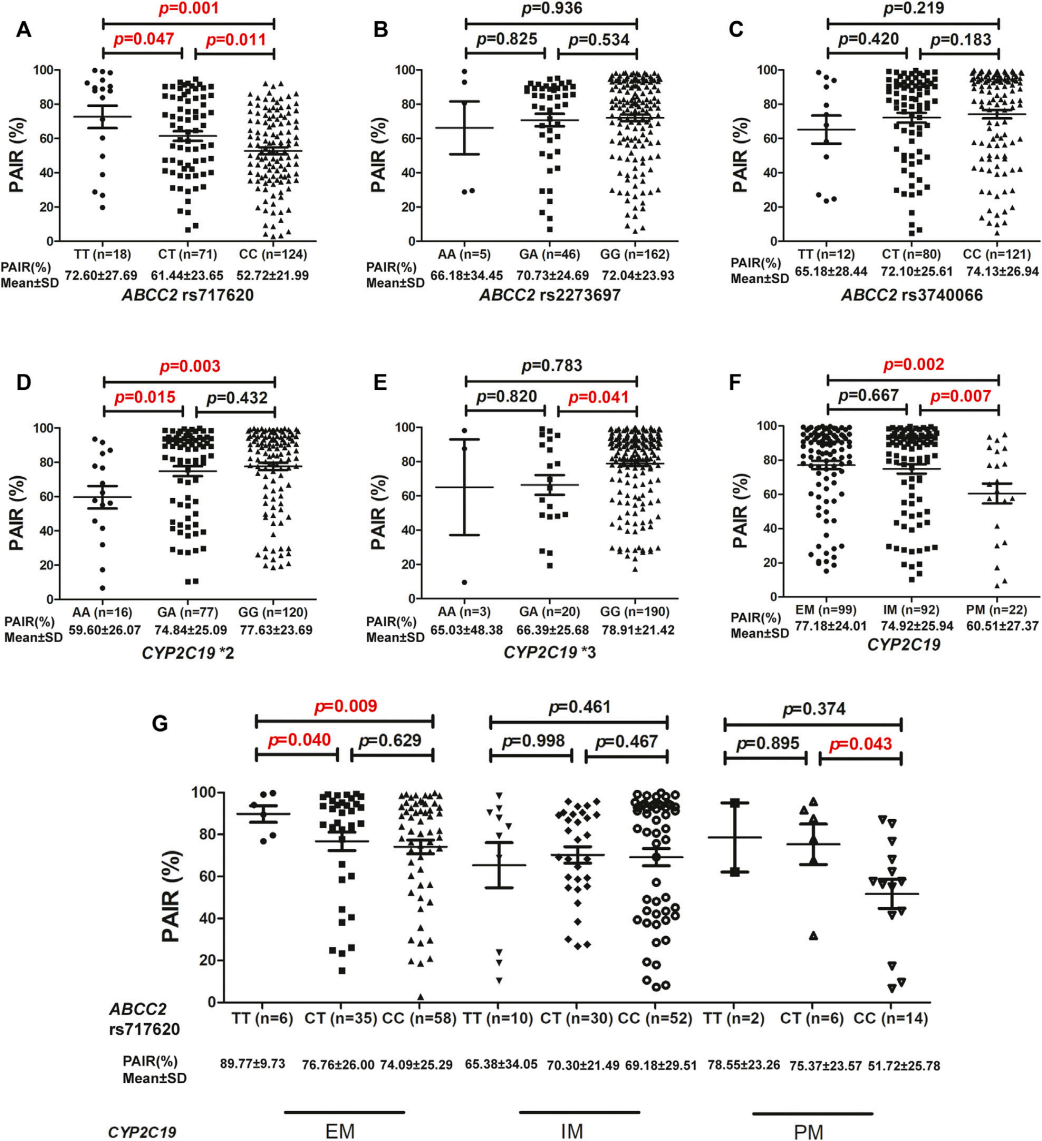
Association between *ABCC2* and *CYP2C19* based on PAIR% values. Association between PAIR% values and **(A)** *ABCC2* rs717620, **(B)** *ABCC2* rs2273697, **(C)** *ABCC2* rs3740066, **(D)** CYP2C19*2, **(E)** CYP2C19*3, and **(F)** *CYP2C19* metabolic phenotype distributions. **(G)** Influence of *ABCC2* rs717620 polymorphism on the PAIR% values of different *CYP2C19* metabolic phenotypes. *PAIR(%),* ADP-induced platelet aggregation inhibition rate.

Correlation analyses were performed to determine the correlation between the three *ABCC2* genetic polymorphisms and the inhibitory effect of clopidogrel on platelet reactivity. Thus, it was observed that *ABCC2* rs717620 TT carriers exhibited significantly higher PAIR% values (72.60 ± 27.69) than both the CT (61.44 ± 23.65) and CC carriers (52.72 ± 21.99) (*p* = 0.047 and *p* = 0.001, respectively). The *ABCC2* rs717620 CT group also presented significantly higher mean PAIR% values (61.44 ± 23.65) than the *ABCC2* rs717620 CC homozygotes (52.72 ± 21.99) (*p* = 0.011) (Figure 1A). However, neither *ABCC2* rs2273697 nor *ABCC2* rs3740066 polymorphisms affected PAIR% values (Figures 1B,C). Additionally, CYP2C19*2 AA carriers presented significantly lower mean PAIR% values (59.60 ± 26.07) than CYP2C19*2 GA carriers (74.84 ± 25.09) (*p* = 0.015). Our results also indicated that CYP2C19*2 AA carriers presented significantly lower mean PAIR% values (59.60 ± 26.07) than CYP2C19*2 GG carriers (77.63 ± 23.69) (*p* = 0.003) (Figure 1D). It was also evident that CYP2C19*3 GA carriers presented significantly lower mean PAIR% values (66.39 ± 25.68) than CYP2C19*3 GG carriers (78.91 ± 21.42) (*p* = 0.041). However, neither CYP2C19*3 AA and GA nor CYP2C19*3 AA and GG carriers presented any significant differences in PAIR% values (Figure 1E). Thus, it was observed that patients with *CYP2C19* PM presented significantly lower PAIR% values (60.51 ± 27.37) than patients with IM (74.92 ± 25.94) or EM (77.18 ± 24.01) (*p* = 0.007 and *p* = 0.002, respectively) (Figure 1F). Based on the *CYP2C19* metabolic phenotype, stratification analysis for the determination of the influence of *ABCC2* rs717620 on PAIR% was further conducted. Conversely, the *ABCC2* rs717620 polymorphism exerted an influence on the PAIR% values corresponding to patients with EM and PM type *CYP2C19*, but not on the values corresponding to patients with IM type *CYP2C19*. Further, in patients with EM type *CYP2C19*, the PAIR% values corresponding to *ABCC2* rs717620 TT carriers were significantly higher (89.77 ± 9.73) than those corresponding to the CT (76.76 ± 26.00) and CC (74.09 ± 25.29) groups (*p* = 0.040 and *p* = 0.009, respectively). Notably, the average PAIR% value of the *CYP2C19* EM group was 77.18, and relatively, when *CYP2C19* EM was combined with *ABCC2* rs717620 TT, the average PAIR% value was 89.77, indicative of a 12.59 increase. When the *CYP2C19* EM group was also combined with *ABCC2* rs717620 CT, the average PAIR% value was 76.76, i.e., it basically remained unchanged. Further, when the *CYP2C19* EM group was combined with *ABCC2* rs717620 CC, the average PAIR% value was 74.09, indicative of a 3.09 decrease. Furthermore, in patients with *CYP2C19* PM, the PAIR% values corresponding to *ABCC2* rs717620 CC carriers (51.72 ± 25.78) were significantly lower than those corresponding to CT carriers (75.37 ± 23.57) (*p* = 0.043). Additionally, the average PAIR% value of the *CYP2C19* PM group was 60.51, and relatively, when the *CYP2C19* PM group was combined with *ABCC2* rs717620 CC, the average PAIR% value was 51.72, indicative of a decrease of 8.79. However, the PAIR% values corresponding to the *ABCC2* rs717620 TT, CT, and CC groups were not significantly different among patients with IM type *CYP2C19* (Figure 1G).

### Relationship between PAIR% values and clinical variables based on stepwise linear regression analysis

Table 2 shows the strength of the relationships between PAIR% values and clinical variables based on stepwise linear regression analysis. The factors that remained significantly correlated with the PAIR% values were *ABCC2* rs717620 (*p* < 0.001), *CYP2C19* *2 (*p* < 0.001), *CYP2C19* *3 (*p* < 0.001), and *CYP2C19* metabolic phenotypes (*p* = 0.022) (Table 2). The removed factors, including *ABCC2* rs2273697, *ABCC2* rs3740066, sex, age, ethnicity (Han Chinese), PLT, MPV, HCY, CHO, TG, HDL-C, and LDL-C showed no significant correlation with the PAIR% values in the stepwise regression analysis. Further, the variance inflation factor (VIF) showed no multicollinearity between the factors (VIF <5), and the PAIR% variability (*R* ^2^) that could be explained by the *ABCC2* rs717620, *CYP2C19* *2, *CYP2C19* *3, and *CYP2C19* metabolic phenotypes was 0.696, 0.506, 0.623, and 0.710, respectively, and 0.852 collectively.

**TABLE 2:** Relationship between PAIR% values and clinical variables based on stepwise linear regression analysis.

Variable	Unstandardized coeffecients		Standardized coeffecients	Collinearity statistics	*p*-value
	B	SE	β	VIF	
Constant	1.463	0.311	—	—	<0.001
*ABCC2* rs717620	0.552	0.041	0.526	1.031	<0.001
CYP2C19*2	−0.327	0.089	−0.533	3.041	<0.001
CYP2C19*3	−0.351	0.069	−0.390	1.605	<0.001
*CYP2C19* metabolic phenotypes	−0.424	0.097	−0.358	3.634	0.022
R2 = 0.852					

B: unstandardized regression coefficient; SE, standard error; β: standardized regression coefficient; VIF, variance inflation factor.

### *ABCC2* single-nucleotide polymorphisms and the risk of low platelet reactivity in patients with coronary artery disease after percutaneous coronary intervention

To investigate whether the *ABCC2* rs717620 gene polymorphism is a risk factor for clopidogrel resistance or hyperreactivity, we performed a multivariate logistic regression analysis after adjusting for the covariates that could potentially affect the PAIR% values, including *ABCC2* rs2273697, *ABCC2* rs3740066, sex, age, ethnicity (Han Chinese), PLT, MPV, HCY, CHO, TG, HDL-C, and LDL-C*.* For *ABCC2* rs717620, three genetic models (additive, dominant, and recessive) were used to analyze the risk of clopidogrel resistance and hyperreactivity. The results showed that, in the three genetic models, *ABCC2* rs717620 remained an independent risk factor for LPR (clopidogrel hyperreactivity) (CC vs. CT: OR, 5.132; 95% CI, 1.855–14.196; *p* = 0.002; CC vs. TT: OR, 9.962; 95% CI, 1.065–93.172; *p* = 0.044; CC vs. CT + TT: OR, 5.076; 95% CI, 1.953–13.193; *p* = 0.001; CC + CT vs. TT: OR, 1.655; 95% CI, 0.173–15.869; *p* = 0.002). Additionally, for CYP2C19*2 and CYP2C19*3, the additive model, out of three genetic model, was found to be adequate based on its metabolism function. CYP2C19*2 also appeared to be a protective factor for LPR (GG vs. GA: OR, 0.044; 95% CI, 0.002–0.970; *p* = 0.048; GG vs. AA: OR, 0.002, 95% CI, 0.001–0.343; *p* = 0.019), and CYP2C19*3 was identified as a major protective factor for LPR (GG vs. GA: OR, 0.041; 95% CI, 0.002–0.717; *p* = 0.029; GG vs. AA: OR, 0.010; 95% CI, 0.005–0.081; *p* = 0.002) (Table 3).

**TABLE 3:** Association between *ABCC2* and *CYP2C19* SNPs and clopidogrel hyperreactivity in 213 patients based on multivariate logistic regression analysis.

Gene/SNP	Genotype	HPR (*n* = 23)	LPR (*n* = 190)	Or (95%CI)	*p* Value	OR^a^ (95%CI)	*p* ^a^ value
*ABCC2*	CC (%)	14 (60.87)	110 (57.89)	1 (ref)		1 (ref)	
rs717620	CT (%)	6 (26.09)	65 (34.22)	4.387 (1.692–11.375)	**0.002**	5.132 (1.855–14.196)	**0.002**
	TT (%)	3 (13.04)	15 (7.89)	8.418 (1.077–70.404)	**0.049**	9.962 (1.065–93.172)	**0.044**
	CC vs. CT + TT			5.599 (1.880–11.250)	**0.001**	5.076 (1.953–13.193)	**0.001**
	CC + CT vs. TT			1.665 (0.187–14.842)	**0.004**	1.655 (0.173–15.869)	**0.002**
CYP2C19*2	GG (%)	14 (60.87)	106 (55.79)	1 (ref)		1 (ref)	
	GA (%)	7 (30.43)	70 (36.84)	0.063 (0.004–0.897)	**0.041**	0.044 (0.002–0.970)	**0.048**
	AA (%)	2 (8.70)	14 (7.37)	0.050 (0.003–0.741)	**0.038**	0.002 (0.001–0.343)	**0.019**
CYP2C19*3	GG (%)	19 (82.61)	171 (90.00)	1 (ref)		1 (ref)	
	GA (%)	3 (13.04)	17 (8.95)	0.063 (0.004–0.897)	**0.014**	0.041 (0.002–0.717)	**0.029**
	AA (%)	1 (4.35)	2 (1.05)	0.005 (0.001–0.008)	**0.014**	0.010 (0.005–0.081)	**0.002**
*CYP2C19* metabolic phenotypes	EM (%)	11 (47.83)	88 (46.32)	1 (ref)		1 (ref)	
	IM (%)	9 (39.13)	83 (43.68)	0.388	0.212 (0.006–7.185)	0.101 (0.003–3.970)	0.221
	PM (%)	3 (13.04)	19 (10.00)	0.125	0.020 (0.001–2.933)	0.007 (0.003–1.563)	**0.042**

The bold value means *p* < 0.05, which was considered statistically significant. SNPs, single nucleotide polymorphisms; LPR: PAIR > 30%, low platelet reactivity; HPR: PAIR ≤ 30%, high platelet reactivity; OR, odds ratio; CI, confidence interval.

a Means-adjusted covariates including ABCC2 rs2273697, ABCC2 rs3740066, sex, age, ethnicity (Han Chinese), PLT, MPV, HCY, CHO, TG, HDL-C, LDL-C. Values in bold are indicative of p < 0.05.

### Hypothetical mechanism

**FIGURE 2:**
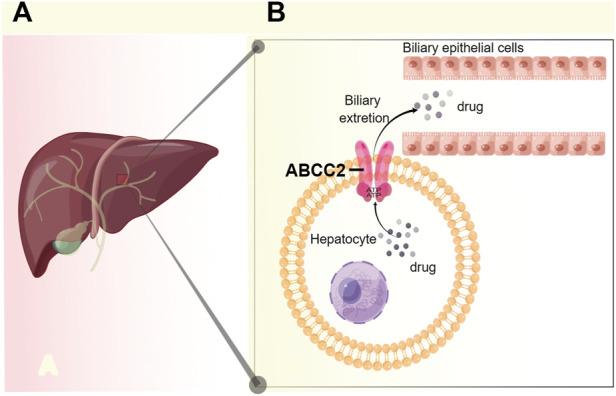
Schematic diagram of the anatomical, cell biological placement, and function of ABCC2 **(A)** In the liver, ABCC2 is localized on the bile canalicular membrane of hepatocytes. **(B)** ABCC2 transport drug by an ATP-dependent manner, and a part of drug is excreted from hepatocyte into bile by ABCC2. Figure adapted from ref. (Higgins, 2007; Toyoda et al., 2016). The figure was drawn using figdraw (https://www.figdraw.com/).

The ABCC2 transporter belongs to the family of ATP-binding cassette (ABC) transporters, in the liver, ABCC2 is localized on the bile canalicular membrane of hepatocytes (Toyoda et al., 2016), which transport substrates through cell membranes by binding and hydrolyzing ATP (Higgins, 2007) (Figure 2).

**FIGURE 3:**
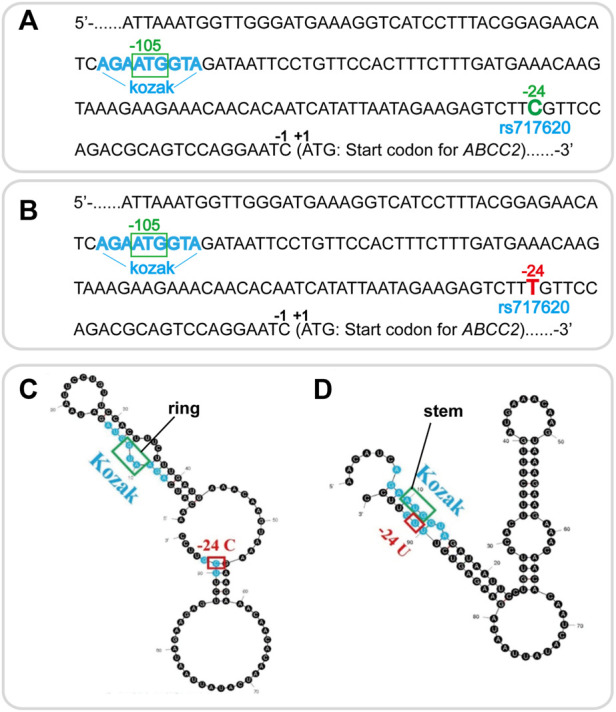
Wildtype and mutation in the mRNA 5′-UTR secondary structure of *ABCC2* rs717620 based on secondary structure modeling.**(A)** cDNA sequence of the 5′UTR part-length of human *ABCC2*. The Kozak sequence is colored blue, and the upstream open reading frame beginning at −105 ATG is shown in the green box. The rs717620 C site at -24 is colored green. **(B)** rs717620 T site at −24 (colored red). **(C)** mRNA sequence of the 5′UTR region of human wildtype *ABCC2* rs717620. In the C allele, the Kozak AUG sequence showed a “ring” structure, enabling Kozak to bind easily to the pre-translation initiation complex. **(D)** Complementary combination of the T allele and the Kozak sequence with AUG on the “stem”, showing the possibility of missing the opportunity for a complementary combination with the AUG of the Kozak sequence, resulting in missed scanning.

The mechanism underlying the effect of *ABCC2* rs717620 on the differential regulation of clopidogrel was investigated. Thus, the cDNA sequence of the 5′UTR part-length of human *ABCC2 rs717620* wild-type and mutant are shown in Figures 3A,B, respectively. Secondary structure modeling by our team revealed a change in the mRNA 5′-UTR secondary structure of *ABCC2*, i.e., the pre-experimental modeling showed that, in rs717620 allele C, the AUG site of the Kozak sequence recognized by the pre-translation initiation complex formed a single chain “ring” structure (Figure 3C). Further, in rs717620 allele T (U in mRNA), U at the -24 site was complementary to the Kozak sequence, forming a hairpin structure; thus, AUG of the Kozak sequence was on the “stem” structure (Figure 3D).

**FIGURE 4:**
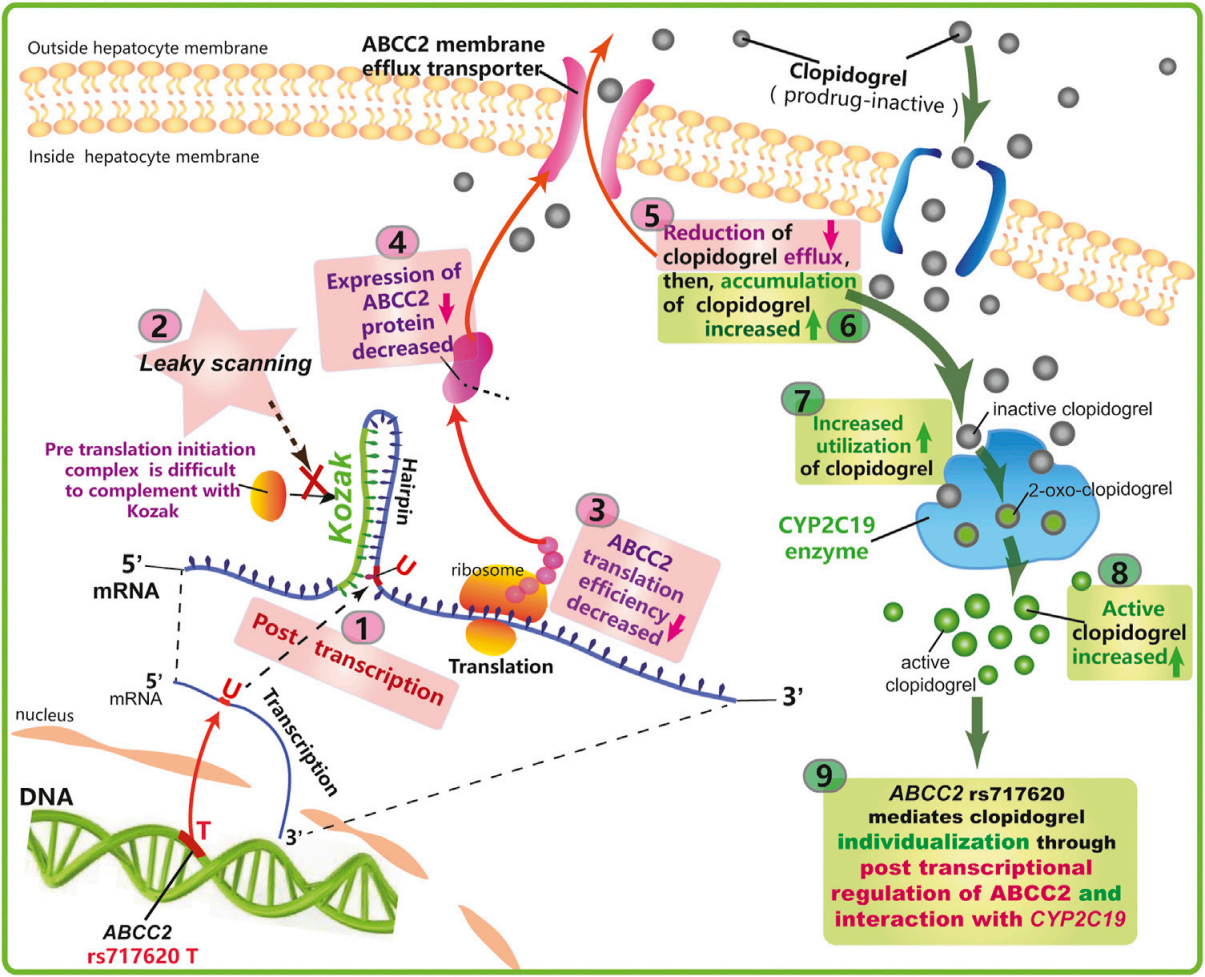
Proposed mechanism underlying the effect of *ABCC2* rs717620 on the differential regulation of clopidogrel.

Based on the function of ABCC2, its anatomical and cell biological placement (Figure 2), and modeling of the mRNA secondary structure (Figure 3), and the association showed in our study (Figure 1; Tables 2,3), we proposed a hypothetical mechanism as follows: *ABCC2* rs717620 possibly mediates individual differences in clopidogrel responses *via* post-transcriptional regulation (Figure 4). The specific steps of the hypothetical mechanism are as follows: *ABCC2* rs717620 is located at position 24 in the 5′-UTR region of the *ABCC2* mRNA, and when *ABCC2* rs717620 T is transcribed to *ABCC2* rs717620 U, the U and Kozak sequences of *ABCC2* complement each other, making it difficult for the transcription initiation complex to complement and combine with AUG, resulting in “leaky scanning”. This leaky scanning of Kozak then significantly reduces the efficiency of ribosomal translation and ABCC2 protein expression. It also reduces the number of ABCC2 efflux transporters at the hepatocyte membrane. Further, a decrease in ABCC2 efflux transporters results in a decrease in the efflux of intracellular clopidogrel out of the plasma membrane, resulting in the increased accumulation and metabolic utilization of the drug. Furthermore, under the action of P450 enzymes, mainly CYP2C19, the amount of clopidogrel converted from inactive clopidogrel to 2-oxo-clopidogrel and then to active clopidogrel increases. This step is also regulated by the *CYP2C19* gene polymorphism.

### A brief summary of this study

**FIGURE 5:**
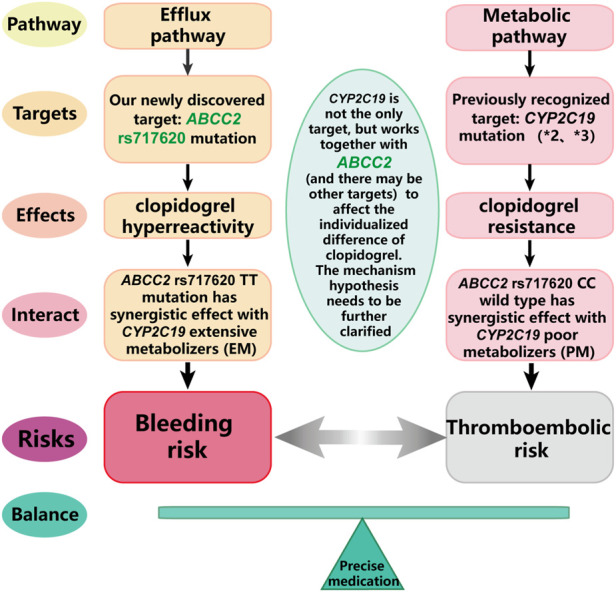
A brief summary of this study.

Taken together, this study showed that only a single *CYP2C19* target cannot guide the use of clopidogrel. *CYP2C19* needs to be detected together with the new target, *ABCC2* rs717620 (and perhaps other unknown targets) to guide medical professionals in clopidogrel precision medicine. Our research group will explore this hypothesis in future mechanistic studies (Figure 5).

## Discussion

ABCC2 is an ATP binding cassette transporter, which is expressed, translocated, and inserted into the apical plasma membrane. Notably, ABCC2 can transfer its substrate, including glucuronide, glutathione, and the sulfate conjugates of several endogenous and exogenous substances, across membranes. The global variability of *ABCC2* includes 27,843 SNPs, such as rs717620, rs2273697, rs3740066, rs927344, rs27843, etc. The global allele frequencies are 13.50, 18.65, 28.81, 0.54, 3.35, respectively in the SNP database of the 1000 Genomes Project Database (www.1000genomes.org).

The altered functioning of *ABCC2* SNPs, especially *ABCC2* rs717620, rs2273697, and rs3740066 can alter the clearance, distribution, and absorption of several clinically important drugs. For example, the *ABCC2* rs717620 T variant is associated with an increased risk of hyperbilirubinemia and mortality in patients with drug-induced liver injury (Huang et al., 2021). It has also been observed that in patients with epilepsy, *ABCC2* rs2273697 and rs3740066 polymorphisms increase blood carbamazepine concentrations (Sha’Ari et al., 2014). Further, the *ABCC2* rs717620 genotype is significantly associated with an increased risk of a decrease in simvastatin dose use (Becker et al., 2013). However, there is no study on the relationship between *ABCC2* and individual differences in clopidogrel response. It is well known that *CYP2C19* plays a critical role in the biological activity of clopidogrel. Further, in patients with loss of function (LOF) alleles (CYP2C19*2 and CYP2C19*3), metabolic transformation slows down, resulting in higher platelet reactivity. Thus, the LOF variation of CYP2C19 affects the response and clinical outcome of clopidogrel. Further, mutations in the *CYP2C19* gene have also been identified as strong predictors of platelet aggregation.

In this study, 213 Chinese patients with coronary heart disease were included and the influence of *ABCC2* gene polymorphisms (rs717620, rs2273697, and rs3740066), CYP2C19*2, and CYP2C19*3 on clopidogrel response were analyzed. Consistent with most previous studies, the five SNP allele frequencies observed were similar to those reported for East-Asian (Han Chinese) subpopulations in the SNP database of the 1000 Genomes Project Database (www.1000genomes.org).

Further, the *ABCC2* rs717620 efflux pathway was found to be closely related to the differential responses to clopidogrel. We also observed that *ABCC2* rs717620 TT carriers exhibited significantly higher PAIR% values and increased risk of clopidogrel hyperreactivity. Thus, to the best of our knowledge, this is the first study to report that *ABCC2* rs717620 gene polymorphism is significantly correlated with individual responses to clopidogrel. Further, carriers of CYP2C19*2 and CYP2C19*3 showed increased risk of clopidogrel resistance. This conclusion is consistent with that of a previous study (Li et al., 2018), and in addition to *CYP2C19*, *N6AMT1* rs2254638 polymorphism also had an effect on clopidogrel resistance in Chinese patients with coronary heart disease. Thus, could be used as an independent biomarker for predicting clopidogrel resistance.

Additionally, *ABCC2* rs717620 brought about significant differences in various metabolic *CYP2C19* types. The difference in PAIR% among the *ABCC2* rs717620 genotypes was observed in both *CYP2C19* EM and PM genotype patients, suggesting that there may be interactions between *ABCC2* rs717620 and *CYP2C19* in the efflux and metabolic utilization of clopidogrel *in vivo*, implying that the TT genotype of *ABCC2* rs717620 and the *CYP2C19* EM metabolic type may jointly lead to low platelet reactivity and clopidogrel hyperreactivity; and that possibly, *ABCC2* rs717620 CC and *CYP2C19* PM work together to bring about high platelet reactivity and clopidogrel resistance. These findings suggests that not only *CYP2C19*, but the *ABCC2* rs717620 of different genotypes also play a role in these patients in determining response to clopidogrel. The specific interaction needs to be studied further.

Stepwise multiple linear regression model showed that there were positive correlations between the *ABCC2* rs717620 with the PAIR%, indicating that an increase in *ABCC2* rs717620 T-allele would be associated with a higher PAIR% and clopidogrel hyperreactivity. In contrast, the negative correlations between the *CYP2C19* *2, *CYP2C19* *3, and *CYP2C19* metabolic phenotypes and PAIR% values indicated that an increase in *CYP2C19* A-allele would be associated with a lower PAIR% value and clopidogrel resistance.

Based on the multivariable-adjusted logistic regression analysis of additive, dominant, and recessive genetic models, we observed that the *ABCC2* rs717620 T allele was an independent risk factor for clopidogrel hyperreactivity. Therefore, in addition to the metabolic pathway for *CYP2C19* utilization, in this study, a new target, *ABCC2* rs717620, of the efflux pathway, which was shown to exert a significant effect on individual responses to clopidogrel, was identified for the first time. Further, there were significant differences between CYP2C19*2 and CYP2C19*3, indicating that CYP2C19*2 and *3 were independent risk factors for clopidogrel resistance. Zhifu et al. (Wang et al., 2019) reported that Han Chinese patients with allele A in CYP2C19*2 and *3 are susceptible to high platelet reactivity and clopidogrel resistance after clopidogrel administration. Consistent with the results of several previous studies, our finding further supported the predictive role of *CYP2C19* polymorphism in clopidogrel efficacy in East-Asian (Han Chinese) ethnic population.

*ABCC2* rs717620 is located on the -24 site of the *ABCC2* 5ʹ-UTR mRNA (Zhang et al., 2010). Further, the role of the Kozak sequence is to combine with the pre-translation initiation complex to improve translation efficiency and protein expression (Li et al., 2018). Therefore, by modeling the *ABCC2* 5ʹ-UTR mRNA secondary structure, we proposed a hypothetical mechanism as follows: *ABCC2* rs717620 may mediate individual differences in response to clopidogrel *via* post-transcriptional regulation. This implies that a decrease in clopidogrel efflux from inside hepatocyte membranes to outside hepatocyte membranes results in its increased accumulation as well as *CYP2C19* metabolic utilization, consequently enhancing the reactivity of clopidogrel. (Kozak, 2005).Further, the hypothesis that *ABCC2* gene polymorphisms affect individual differences in clopidogrel reactivity *via* changes in mRNA secondary structure, which needs to be investigated further, provides a new direction for future research. Several studies have shown that *ABCC2* SNPs mediate the efflux of irinotecan, cisplatin, and methotrexate from intracellular to extracellular spaces in colorectal cancer, ovarian cancer, and acute lymphoblastic leukemia (Corpechot et al., 2020; Huang et al., 2021). Therefore, this hypothetical mechanism may also provide novel ideas regarding *ABCC2* that will facilitate studies on the mechanism of other drugs in other diseases.

This study had some limitations. First, the rapid allele CYP2C19*17 was not genotyped (Sim et al., 2006), which would mis-classify the normal phenotype. This would be genotyped in our future research. Second, clinical information on comorbidity and concomitant medication were not all collected. This would be considerd in our future studies. Third, the clinical outcomes of the patients included in this study was not considered. This would also be supplemented in our future research. Fourth, the exact mechanisms were not comprehensively investigated. Thus, further studies are still needed in this regard.

## Conclusion

Based on the results of this study, we proposed that the ABCC2 efflux pathway affects individual responses to clopidogrel, and also suggested a novel potential target, *ABCC2* rs717620, i.e., our results confirmed that *ABCC2* rs717620 as well as CYP2C19*2 and *3 are related to individual responses to clopidogrel, and there may be interactions between *CYP2C19* and *ABCC2* rs717620 metabolic types. Moreover, the TT of *ABCC2* rs717620 was found to be an independent risk factor for clopidogrel hyperreactivity, while CYP2C19*2 and *3 were found to be independent risk factors for clopidogrel resistance. Further, preliminary mechanistic studies suggested that *ABCC2* rs717620 alters the secondary structure of mRNA and causes leaky Kozak scanning. This post-transcriptional regulation may be the reason for the individual differences in clopidogrel repsonse; however, further indepth studies are still needed in this regard. We hope that the results of this study provide insights into new targets to facilitate personalized decision making regarding clopidogrel therapy. Moreover, further research on the hypothetical mechanism by which *ABCC2* affects individualized difference in clopidogrel response may also provide new ideas regarding the mechanism of individualized differences in responses to other drugs dependent on ABCC2.

## Data Availability

The original contributions presented in the study are included in the article/Supplementary Material, further inquiries can be directed to the corresponding authors.
