# Supplementary material for: Association of ABCC2 polymorphism with clopidogrel response in Chinese patients undergoing percutaneous coronary intervention
Source: Front Pharmacol. 2022 Oct 7;13:889473. doi: 10.3389/fphar.2022.889473 (PMC9585281; doi:10.3389/fphar.2022.889473)
Supplement: Supplementary file 1 [file Table1.docx]

## Supplemental Table 1. The allelic frequencies of the *ABCC2* and *CYP2C19* SNPs in our study.

| **Gene SNP (n = 213)** | **Allele Frequency (%) of our study (n = 213)** | | **Allele frequency (%) in the 1000 Genomes database**  **(East Asian - Han Chinese) (n = 1008)** | | ***p*-value** |
| --- | --- | --- | --- | --- | --- |
| ***ABCC2* rs717620** | **C** | **T** | **C** | **T** |  |
|  | 74.88 | 25.12 | 78.27 | 21.73 | 0.248 |
| ***ABCC2* rs2273697** | **G** | **A** | **G** | **A** |  |
|  | 86.85 | 13.15 | 90.38 | 9.62 | 0.123 |
| ***ABCC2* rs3740066** | **C** | **T** | **C** | **T** |  |
|  | 75.59 | 24.41 | 75.30 | 24.70 | 0.929 |
| ***CYP2C19**2** | **G** | **A** | **G** | **A** |  |
| (rs4244285) | 74.41 | 25.59 | 68.75 | 31.25 | 0.117 |
| ***CYP2C19**3** | **G** | **A** | **G** | **A** |  |
| (rs4986893) | 93.90 | 6.10 | 94.44 | 5.56 | 0.753 |
